# Direct DME synthesis on CZZ/H-FER from variable CO_2_/CO syngas feeds†

**DOI:** 10.1039/d0ra09754c

**Published:** 2021-01-12

**Authors:** Stefan Wild, Sabrina Polierer, Thomas A. Zevaco, David Guse, Matthias Kind, Stephan Pitter, Karla Herrera Delgado, Jörg Sauer

**Affiliations:** IKFT – Institute of Catalysis Research and Technology, Karlsruhe Institute of Technology Hermann-von-Helmholtz-Platz 1, D-76344 Eggenstein-Leopoldshafen Germany karla.herrera@kit.edu stephan.pitter@kit.edu; TVT – Institute of Thermal Process Engineering, Karlsruhe Institute of Technology Kaiserstraße 12 D-76131 Karlsruhe Germany

## Abstract

Catalyst systems for the conversion of synthesis gas, which are tolerant to fluctuating CO/CO_2_ gas compositions, have great potential for process-technical applications, related to the expected changes in the supply of synthesis gas. Copper-based catalysts usually used in the synthesis of methanol play an important role in this context. We investigated the productivity characteristics for their application in direct dimethyl ether (DME) synthesis as a function of the CO_2_/CO_*x*_ ratio over the complete range from 0 to 1. For this purpose, we compared an industrial Cu/ZnO/Al_2_O_3_ methanol catalyst with a self-developed Cu/ZnO/ZrO_2_ catalyst prepared by a continuous coprecipitation approach. For DME synthesis, catalysts were combined with two commercial dehydration catalysts, H-FER 20 and γ-Al_2_O_3_, respectively. Using a standard testing procedure, we determined the productivity characteristics in a temperature range between 483 K and 523 K in a fixed bed reactor. The combination of Cu/ZnO/ZrO_2_ and H-FER 20 provided the highest DME productivity with up to 1017 g_DME_ (kg_Cu_ h)^−1^ at 523 K, 50 bar and 36 000 ml_N_ (g h)^−1^ and achieved DME productivities higher than 689 g_DME_ (kg_Cu_ h)^−1^ at all investigated CO_2_/CO_*x*_ ratios under the mentioned conditions. With the use of Cu/ZnO/ZrO_2_//H-FER 20 a promising operating range between CO_2_/CO_*x*_ 0.47 and 0.8 was found where CO as well as CO_2_ can be converted with high DME selectivity. First results on the long-term stability of the system Cu/ZnO/ZrO_2_//H-FER 20 showed an overall reduction of 27.0% over 545 h time on stream and 14.6% between 200 h and 545 h under variable feed conditions with a consistently high DME selectivity.

## Abstract 2

Catalyst systems for the conversion of synthesis gas, which are tolerant to fluctuating CO/CO_2_ gas compositions, have great potential for process-technical applications, related to the expected changes in the synthesis gas supply.

## Graphical abstract

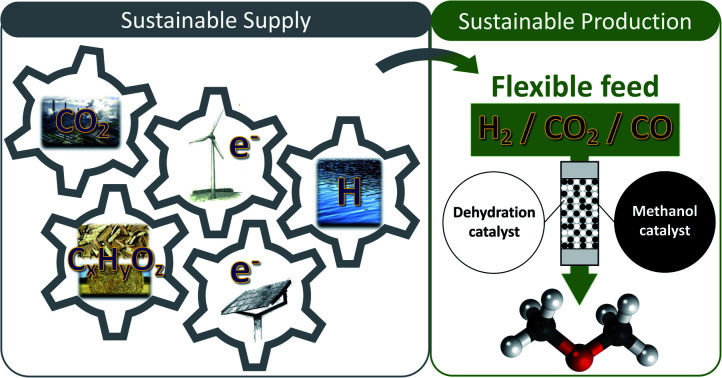

## Introduction

Power-to-fuels concepts play a major role for the future integration of carbon neutral technologies within complex energy supply systems.^1,2^ Amongst potential non-fossil carbon resources for the production of synthetic hydrocarbons, carbon dioxide plays a dominant role. Once used in combination with sustainable, economically viable hydrogen production, CO_2_ would allow the production of carbon neutral fuels and industrial chemicals^3^ and, on the other hand, contribute to a mitigation of its environmental impact.^4^ In particular, the foreseeable dynamic character in power generation demands the development of robust processes that enable highly adaptive operation modes. A flexible production of chemical energy carriers from CO_2_-rich syngas, catalysed by efficient and long-term stable catalysts is hereby one of the most promising options. Besides other synthetic hydrocarbon-based energy carriers, dimethyl ether (DME) is a particularly interesting candidate due to its promising physical and chemical properties.^5–7^ It can be either directly used as diesel substitute^8^ or as intermediate for the production of a wide range of synthetic hydrocarbons.

The DME synthesis is technically feasible in a one-step (*i.e.* reactions (R1) to (R4) in a single reactor)^9–14^ or two-step process (*i.e.* reactions (R1) to (R3) in one reactor, and reaction (R4) in a second reactor),^15–18^ typically using a Cu/ZnO-based catalyst (*e.g.* Cu/ZnO/Al_2_O_3_) for MeOH formation and a solid-acid catalyst such as γ-Al_2_O_3_, silica-modified alumina or zeolites for MeOH dehydration to DME. Compared to the industrially applied two-step process, the direct process allows higher CO_*x*_ conversion and a simplified reactor design resulting in reduced investment costs.^9–13^ In both processes, catalyst productivity strongly depends on the syngas composition, *i.e.* the ratios between H_2_, CO and CO_2_.^19,20^ Theoretical studies suggest that the synergistic effect of Cu and Zn containing domains in the MeOH forming catalyst is largely dependent on the feed composition.^21^ Also *in situ* investigations^22,23^ showed that changes in the catalytic activity of Cu/ZnO-based catalysts are caused by altered syngas composition leading to reversible changes of the catalyst morphology during MeOH formation from CO and CO_2_ hydrogenation.

CO hydrogenation to MeOH

R1 CO + 2H_2_ ⇌ CH_3_OH Δ*H*° 298 *K* = −90.4 kJ mol^−1^

CO_2_ hydrogenation to MeOH

R2 CO_2_ + 3H_2_ ⇌ CH_3_OH + H_2_O Δ*H*° 298 *K* = −49.4 kJ mol^−1^

Water-gas shift (WGS) and its reverse reaction (rWGS)

R3 CO + H_2_O ⇌ CO_2_ + H_2 _Δ*H*° 298 *K* = −41.0 kJ mol^−1^

MeOH dehydration

R4 2CH_3_OH ⇌ CH_3_OCH_3_ + H_2_O Δ*H*° 298 *K* = −23.5 kJ mol^−1^

The use of CO_2_ as co-feed in the direct DME synthesis has been encouraged, however, this brings additional challenges predominantly associated with loss of catalyst activity,^19,20,24,25^ since additional water is formed through reaction (R2) and (R3). This challenge requires robust catalytic systems, particularly with higher water tolerance.^24,26–28^ Catalytic systems enabling both, CO and CO_2_ hydrogenation should therefore be equipped with a dehydration component with sufficient acidity for effective MeOH dehydration and concurrently, with appropriate hydrophobic surface characteristics to reduce the adsorption of water.^29,30^

Although Cu/ZnO/Al_2_O_3_ (CZA) catalysts are highly active and selective for MeOH synthesis from CO/H_2_, their activity towards CO_2_ hydrogenation is reduced.^31–33^ Amongst several alternative catalytic systems studied, it was proposed to improve CO_2_ conversion by using less hydrophilic promoters, such as ZrO_2_ instead of Al_2_O_3_.^34–36^ A large number of publications on the direct DME synthesis refer to the conversion of either CO or CO_2_ as the sole carbon source.^25,37^ However, the use of CO-pure syngas promotes coke formation,^38^ catalyst deactivation^39,40^ and CO_2_ formation, whereas CO_2_-pure syngas increases H_2_ requirement, water formation and lowers thermodynamic equilibrium.^41^ Consequently, a logical trade-off seems to be a syngas mixture involving CO and CO_2_. Although the issue of variable CO/CO_2_ feed compositions has been addressed in some previous studies, no truly satisfactory catalytic system has been thoroughly investigated for a wide variation range of CO/CO_2_ in combination with its long-term stability.^19,42–45^

Recently we showed that a novel continuous co-precipitation process leads to a Cu/ZnO/ZrO_2_ (CZZ) catalyst, which in combination with a ferrierite dehydration co-catalyst shows improved productivity for DME.^46^

The scope of our work is to investigate the tolerance of different catalytic systems, especially CZZ/FER, to variable changes in process parameters, particularly the influence of the volumetric CO_2_/CO_*x*_ inlet-ratio on DME productivity, with the aim of simultaneously maintaining productivity at a high level over a longer period of time. To understand the interplay of the MeOH forming catalyst with the MeOH dehydrating catalyst depending on the syngas feed composition, we compared two dehydration catalysts, γ-Al_2_O_3_, which is known to offer high DME productivity in CO-rich feeds while the formation of olefins is inhibited, due to its low acidity,^47^ and a FER-type zeolite with increased Brønsted acidity, having shown a reasonable water tolerance in the direct DME synthesis from CO_2_.^48^

Our hypothesis is that in this way it will be possible to determine what are the appropriate operating parameters under which reasonable DME production with a variable syngas composition takes place.

## Experimental

### Catalyst preparation

The CZZ catalyst was prepared by continuous co-precipitation method from metal nitrate solution and sodium bicarbonate at pH 7 using a micro jet mixer. The resulting solution was aged at 313 K for 2 h. The precipitate was filtered, dried at 383 K for 16 h and calcined at 623 K with 3 K min^−1^ for 4 h. The method was described in detail by Polierer *et al.*^46^

A commercial CZA catalyst was used for comparison purposes. Commercial γ-Al_2_O_3_ (Alfa Aesar) or a ferrierite-type zeolite H-FER 20 (FER) (Zeolyst International) were used as dehydration catalysts. Before use, FER was calcined at 823 K for 4 h in air.

For activity tests all catalyst components were finely powdered, pressed and sieved into sieve fractions of 250–500 μm and then physically mixed with a mass ratio of 1 : 1 resulting in three catalytic systems: CZA/FER, CZZ/γ-Al_2_O_3_ and CZZ/FER. Since reactions (R1) to (R4) are exothermic, the catalysts were diluted with silicon carbide (SiC, Hausen Mineraliengroβhandel GmbH) with the same grain size in a mass ratio of 1 : 10 in order to minimize hot spot formation and therefore ensure largely isothermal operation.

### Catalyst characterization

For a detailed characterization of the CZZ and the commercial CZA pre-catalysts we refer to our recent study.^46^ Selected properties of the MeOH pre-catalysts are shown in Table 1. Physico-chemical properties of the commercial acid dehydration catalysts are taken from Kim *et al.*^49^ and shown in Table 2.

**Table 1:** Selected pre-catalyst properties of CZZ and com. CZA taken from Polierer *et al.*^46^

Catalyst	Cu/wt%	Zn/wt%	Zr/wt%	Al/wt%	*S*_BET_/m^2^ g^−1^	*S*_Cu_/m^2^ g^−1^	*d*_CuO_/nm calcined catalyst	*d*_CuO_/nm spent catalyst
CZZ	61	31	8	—	125	27	4	10
Com. CZA	64	29	—	6	98	13	4	8

**Table 2:** BET surface and total acidity properties of the acid dehydration catalysts γ-Al_2_O_3_ and FER at low-temperature (LT) and high-temperature (HT) taken from Kim *et al.*^49^

Catalyst	NH_3_-TPD peak position/°C			Acid amount/mmol NH_3_ per g_cat_		
	*S*_BET_/m^2^ g^−1^	LT region	HT region	Total acidity	LT region	HT region
γ-Al_2_O_3_	213	239	351	0.37	0.18	0.19
FER	390	208	383	0.70	0.31	0.39

### Activity tests

**Fig. 1:**
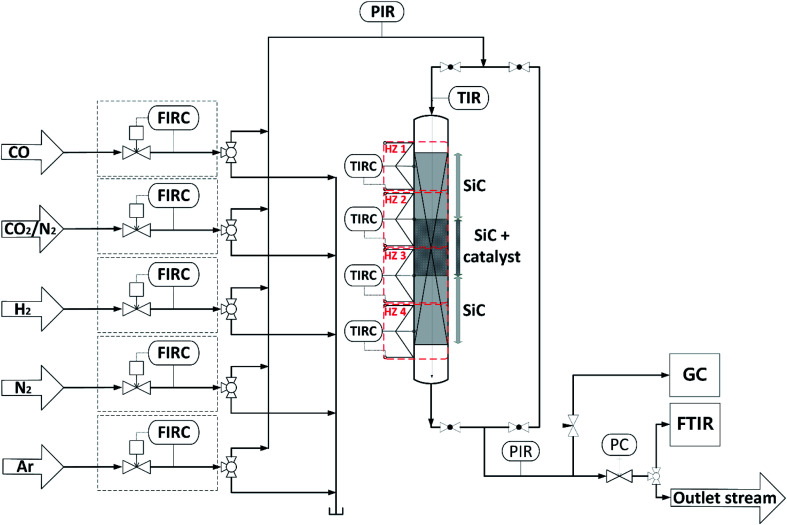
Schematic flowchart of the experimental setup used for the investigation of the direct DME synthesis.

Direct DME synthesis was performed in a stainless steel fixed bed reactor with an inner diameter of 12 mm and a length of 460 mm, filled with a physical mixture of 2 g admixed catalyst and 20 g SiC. The reactor was heated by four independent heating zones depicted in Fig. 1, to ensure an axial temperature difference within the catalyst bed of typically less than 2 °C. The gas supply was controlled using mass flow controllers (Bronkhorst Hi-Tec). Feed gases, carbon monoxide (CO, 99.97%), argon (Ar, 99.9999%), nitrogen (N_2_, 99.9999%), hydrogen (H_2_, 99.9999%) and a mixture carbon dioxide/nitrogen (CO_2_/N_2_, 50 : 50 ± 1.0 vol%) were provided by Air Liquid Germany GmbH. Product gas composition was analyzed by a gas chromatograph (Agilent G1530A), equipped with thermal conductivity (TCD) and flame ionization (FID) detectors connected to RT®-U-BOND and RT®-Molecular sieve 5A columns. Volumetric water concentration was determined with a FTIR CX4000 (Gasmet Technologies GmbH). Reduction of CZA and CZZ catalyst was performed at 1 bar with 5 vol% H_2_ diluted in Ar, while temperature was increased from 373 K to 473 K with a ramp of 20 K h^−1^, followed by further heating to a final reduction temperature of 513 K with 50/50 vol% H_2_/Ar at a rate of 12 K h^−1^. Reduction temperature was kept for another 5 h, before the reactor was purged with Ar and cooled to 493 K. Subsequently, the pressure was increased to 50 bar to perform direct DME synthesis. Feed gas compositions used are shown in Table 3. As CO_2_ hy`drogenation to MeOH (R2) requires stoichiometrically 1.5 equivalents more H_2_ than CO hydrogenation (R1), the H_2_ content was adjusted along different CO_2_/CO_*x*_ inlet-ratios according to (1).

1 *y*_H2,in_ = 2.3 (*y*_CO_2_,in_ + *y*_CO,in_) + *y*_CO_2__,_in_

**Table 3:** CO_2_/CO_*x*_ inlet-ratios and respective feed gas compositions used in direct DME synthesis

CO_2_/CO_*x*_	H_2_/vol%	CO/vol%	CO_2_/vol%	N_2_/vol%	Ar/vol%
0.00	34.5	15.0	0.0	15.0	35.5
0.07	35.5	14.0	1.0	15.0	34.5
0.20	37.5	12.0	3.0	15.0	32.5
0.47	41.5	8.0	7.0	15.0	28.5
0.80	46.5	3.0	12.0	15.0	23.5
1.00	49.5	0.0	15.0	15.0	20.5

Each feed gas composition was investigated at five temperatures between 483 and 523 K and two gas-hourly space velocities (GHSV) of 18 000 and 36 000 ml_N_ (g h)^−1^ with regard to the mass of Cu-based catalyst.

The general sequence for the process parameters variation is shown in Fig. S1†. After finishing the variation loops of CO_2_/CO_*x*_ values for each temperature, the reactor was purged with Ar for two hours, followed by setting a chosen reference point of 18 000 ml_N_ (g h)^−1^, 503 K and CO_2_/CO_*x*_ inlet-ratio of 0.8 at 50 bar. Repeated measurements at the reference point were performed to monitor catalyst stability.

### Indexes of performance

In all experiments, the carbon balance presented a maximum deviation of ±3%, calculation were performed using eqn S1†. The performance indicators were calculated as follows:

CO_*x*_ conversion:2
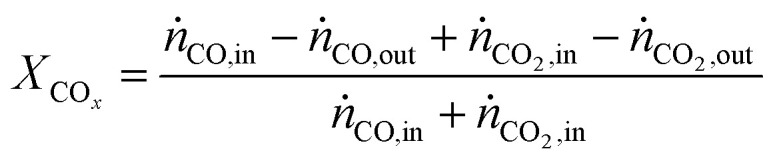


Cu-mass-specific DME/MeOH productivies:3
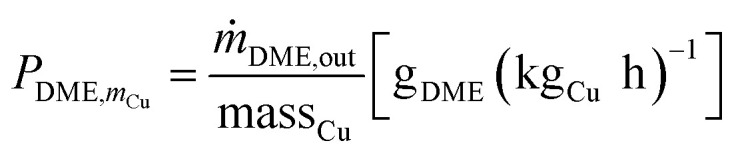
4
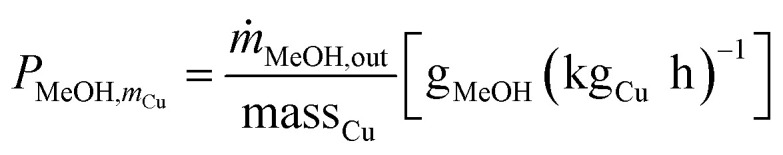


In order to show the influence of the CO_2_/CO_*x*_ ratio on CO and CO_2_ hydrogenation, each in their role (*i.e.* reactant or product) on DME and MeOH formation three different cases were defined for the selectivity calculation.

#### Case 1: CO; CO_2_: reactants

CO and CO_2_ are converted, which results in the CO_*x*_-based selectivity calculation (5):5
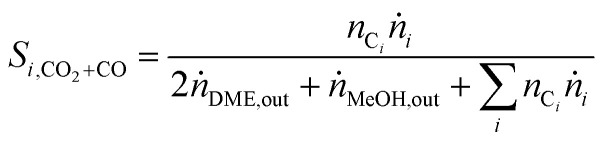
where *n*_C_*i*__ corresponds to the number of carbon atoms in each product and *ṅ*_*i*_ to the respective molar flowrate.

#### Case 2: CO: reactant; CO_2_: product

CO is converted while CO_2_ is a product. Here selectivity (6) is defined as follows:6
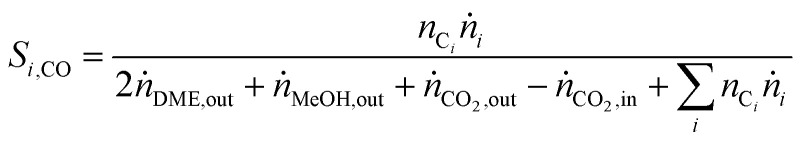


#### Case 3: CO_2_: reactant; CO: product

CO_2_ is converted while CO is a product. Here selectivity (7) is defined as follows:7
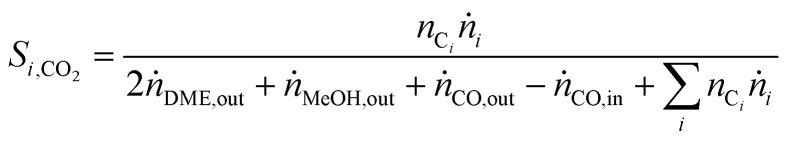


## Results and discussion

### Comparison of γ-Al_2_O_3_ and FER as dehydration catalysts

**Fig. 2:**
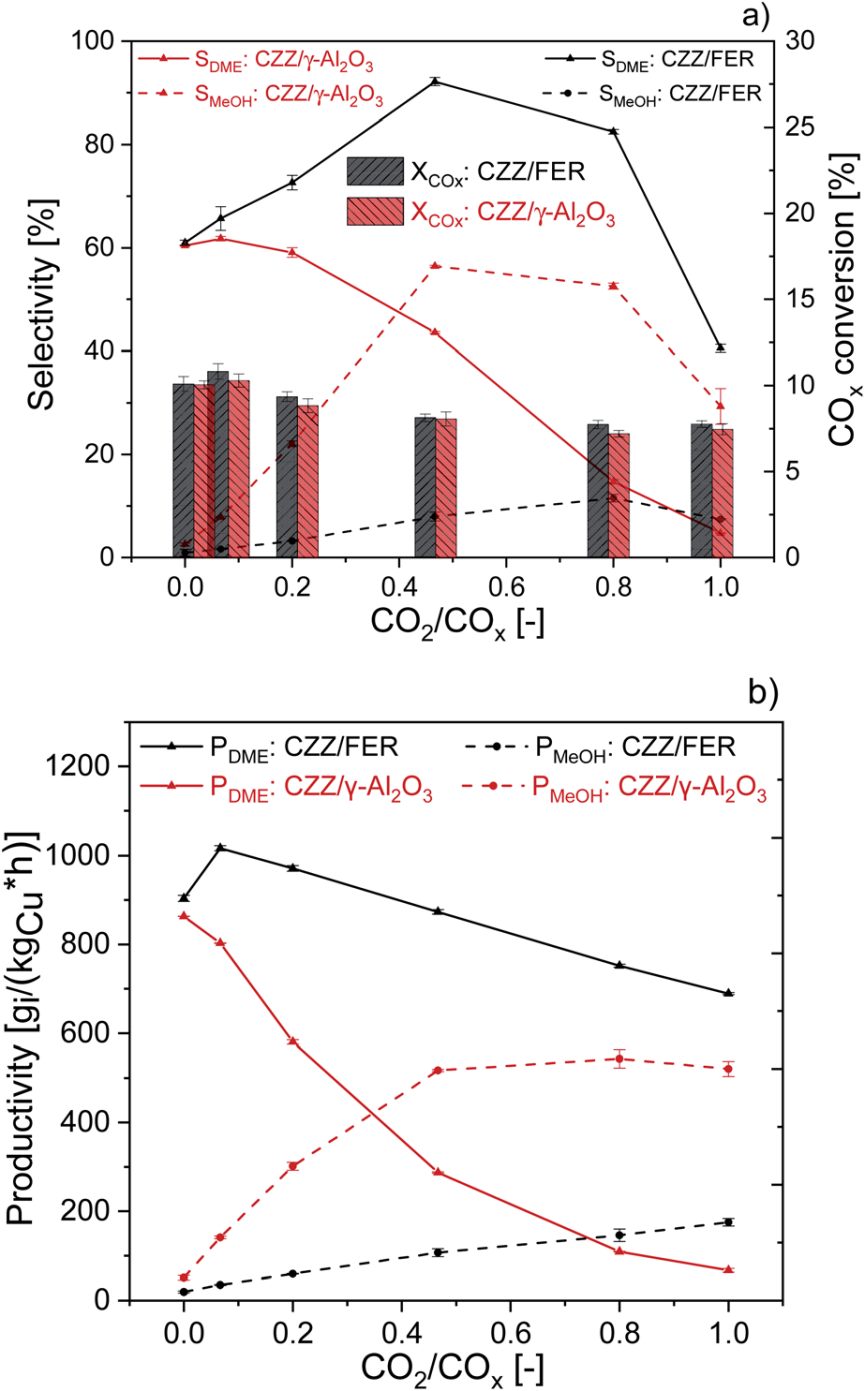
Influence of the CO_2_/CO_*x*_ inlet-ratio on direct DME synthesis with CZZ/FER (1 : 1 wt%) (black) and CZZ/γ-Al_2_O_3_ (1 : 1 wt%) (red) at 50 bar, 523 K and 36 000 ml_N_ (g h)^−1^. (a) CO_*x*_ conversion (right axis) and selectivities of MeOH and DME (left axis). (b) Productivities of MeOH and DME.

In Fig. 2, CZZ/γ-Al_2_O_3_ is compared to CZZ/FER at different CO_2_/CO_*x*_ inlet-ratios with regard to CO_*x*_ conversion (Fig. 2a, bars), selectivities to MeOH and DME (Fig. 2a, lines) and, productivities of MeOH and DME (Fig. 2b). Regarding the CO_*x*_ conversion (Fig. 2a), a slight increase of CZZ/FER in comparison to CZZ/γ-Al_2_O_3_ is observable. As the MeOH catalyst is the same in both systems, this difference is attributed to the dehydration catalysts. Since the DME selectivity of CZZ/FER is higher than the one of CZZ/γ-Al_2_O_3_, there is an increased intermediate product (MeOH) removal with CZZ/FER. Consequently, there is an increase in MeOH production due to an equilibrium shift of the CO and CO_2_ hydrogenation ((R1) and (R2)), resulting in a slightly higher CO_*x*_ conversion. As the CO_*x*_ conversion is strongly kinetically controlled under the respective operating conditions (Fig. S2†) the enhancement due to equilibrium shift is only slightly pronounced. The CZZ/FER system reaches its highest DME selectivity of 92.1% at a CO_2_/CO_*x*_ inlet-ratio of 0.47, and even at 0.8 selectivity is still above 80%. CZZ/γ-Al_2_O_3_ shows a reduced DME selectivity up to 60% at CO_2_/CO_*x*_ inlet-ratios below 0.2, a further increase of the CO_2_ content leads to a strongly declining DME selectivity with a minimum of 4.8% at CO_2_/CO_*x*_ inlet-ratio of 1.00. Accordingly, CZZ/FER generally achieves higher DME productivity, with the difference to CZZ/γ-Al_2_O_3_ becoming more noticeable at higher CO_2_/CO_*x*_ inlet-ratios (Fig. 2b). Interestingly, CZZ/FER already enables a significantly improved DME productivity (67%) compared to CZZ/γ-Al_2_O_3_ at a relatively low CO_2_/CO_*x*_ inlet-ratio of 0.20, what can be attributed to the strong hydrophilic behaviour of γ-Al_2_O_3_ as reported in literature.^12,19,33,50^ On the other hand FER is marked by better water resistance, it has a higher acidity compared to γ-Al_2_O_3_ (see Table 2) and additionally well distributed acid sites with a suitable strength and a good resistance to coke formation in the presence of water,^12,30,33,51^ and therefore is superior for dehydration of MeOH formed at high CO_2_ content. The nearly constant CO_*x*_ conversion (Fig. 2a) as well as the improved DME selectivity of CZZ/FER compared to CZZ/γ-Al_2_O_3_ (Fig. 2b) lead to a superior DME productivity between 1017 g_DME_ (kg_Cu_ h)^−1^ (CO_2_/CO = 0.20) and 689 g_DME_ (kg_Cu_ h)^−1^ (CO_2_/CO_*x*_ = 1.00). Due to the high DME productivities at variable CO_2_/CO_*x*_ feed compositions, FER was chosen for further investigations.

### Comparison of MeOH catalysts CZA and CZZ

**Fig. 3:**
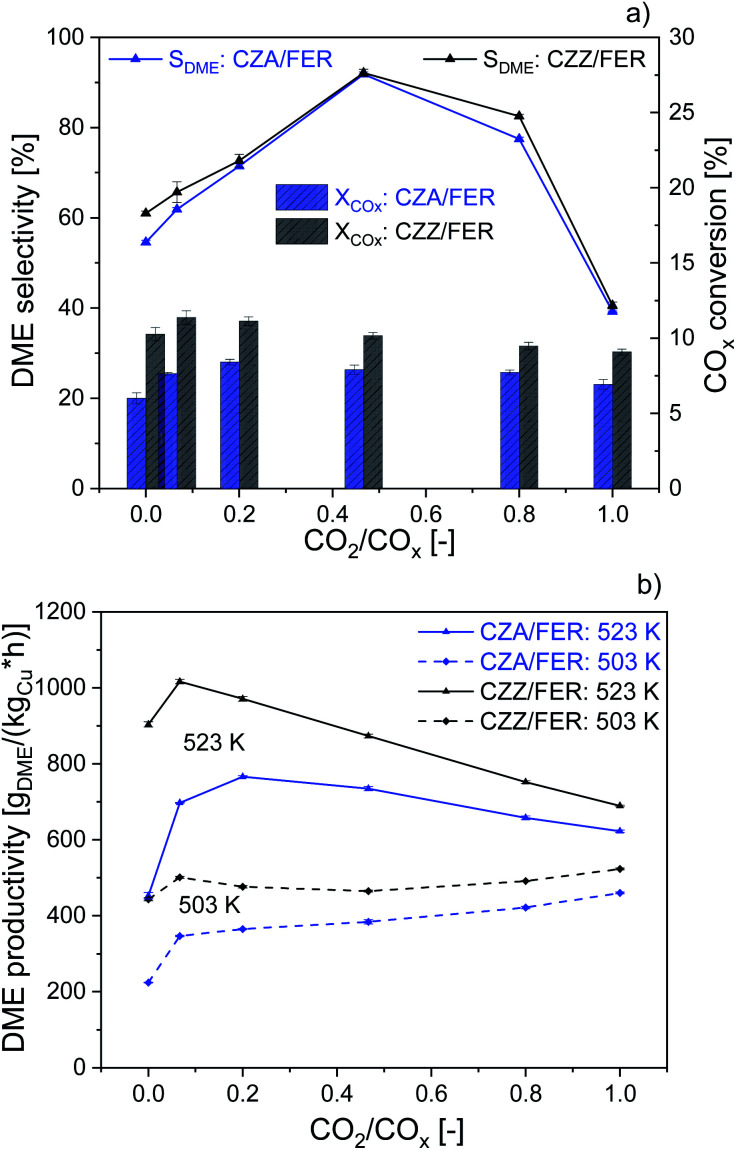
Influence of CO_2_/CO_*x*_ inlet-ratio on the DME synthesis with com. CZA catalyst (blue) and the CZZ catalyst (black) at 50 bar and GHSV: 36 000 ml_N_ (g h)^−1^, mixed with FER (1 : 1 wt%). DME selectivity and CO_*x*_ conversion ((a) 523 K). DME productivity ((b) 503 and 523 K).

We further studied the catalytic activity of the self-prepared CZZ and a commercial CZA catalyst as a benchmark, which is typically used for MeOH synthesis from CO-rich syngas, both in combination with FER. In Fig. 3 we compare CZA/FER (blue) and CZZ/FER (black) at different CO_2_/CO_*x*_ inlet-ratios. Fig. 3a displays CO_*x*_ conversion (bars) and selectivities to MeOH and DME (lines) at 523 K, while Fig. 3b represents the productivities of MeOH and DME at 503 and 523 K. CZZ/FER enables significantly elevated CO_*x*_ conversion for all investigated CO_2_/CO_*x*_ inlet-ratios compared to CZA/FER (Fig. 3a), resulting in correspondingly higher DME productivity values (Fig. 3b). We attribute the enhanced CO_*x*_ conversion to the properties of the continuously co-precipitated CZZ, *i.e.* its high Cu surface area (Table 1) and the presence of ZrO_2_, which is known to promote Cu dispersion^11^ and increase the activity of Cu-based catalysts in CO_2_ hydrogenation to MeOH and DME.^11,35,46,52^ It is interesting to note that although CZA has a relatively low copper surface area of 13 m^2^ g^−1^ CZA/FER offers high DME productivities: with pure H_2_/CO_2_ (according to CO_2_/CO_*x*_ = 1) at 523 K, the productivity is only 9% lower than using CZZ/FER (CZZ-*S*_Cu_: 27 m^2^ g^−1^).

Similar observations were made by Kurtz *et al.*^53^ showing a pronounced linear dependence of MeOH activity and *S*_Cu_ using Cu/ZnO catalysts, whereby using a self-prepared CZA the MeOH activity increased non-linearly to copper surface area. Moreover, uncharacterized additional components of the commercial CZA could also influence its activity. Fig. 3a shows that this increased DME productivity in Fig. 3b is caused by higher CO_*x*_ conversion and DME selectivity. This observation is consistent with results presented in the literature.^54–56^ According to Behrens *et al.*^57^ and Studt *et al.*,^21^ CO_2_ hydrogenation (R2) is significantly faster than CO hydrogenation (R1) on a Cu/ZnO-based catalyst. Therefore, with additional CO_2_ in the feed, MeOH formation takes place more quickly at the beginning of the catalyst bed, whereas with a pure H_2_/CO feed, CO_2_ hydrogenation is only accelerated when part of the DME has already been produced and additional CO_2_ is generated *via* the WGS with the water formed in the process. By lowering the reactor temperature (503 K), the DME productivity of both catalyst systems changes only slightly over the entire CO_2_/CO_*x*_ feed range. With CZZ/FER DME productivity ranges between 433 and 523 g_DME_ (kg_Cu_ h)^−1^. We consider this to be a combination of different effects: firstly, a reduced rate of endothermic rWGS (R3) results in less water being formed, which is able to inhibit the activity of the admixed catalyst,^58^ and secondly the positive effect on the thermodynamic equilibrium of CO_*x*_ conversion (R1), (R2) and MeOH dehydration (R4). Similar observations have been made by Sahibzada *et al.*^59^ using a CZA catalyst for MeOH synthesis, by increasing the CO_2_/CO_*x*_ inlet-ratio a continuously increasing MeOH productivity takes place as long as differential conditions prevail. The benefit of a slight increase of CO_2_ in feed (CO_2_/CO_*x*_ inlet-ratio from 0.00 to 0.07) leads to a maximum in DME productivity of 1017 g_DME_ (kg_Cu_ h)^−1^ using CZZ/FER at 523 K. The DME productivity of CZZ/FER then gradually decreases to 689 g_DME_ (kg_Cu_ h)^−1^ using CO_2_ as the sole carbon source, which we regard as an important argument for process operation with dynamically variable feed compositions. An increasing CO_2_ content changes the thermodynamic equilibrium,^41^ increases water formation and leads to a more oxidative atmosphere – resulting in a change of the Cu/Zn and Cu sites^23,60^ which negatively affects CO_2_ hydrogenation – leading to a performance levelling of the two catalyst systems in terms of CO_*x*_ conversion and DME productivity. Frusteri *et al.*^12^ investigated admixed catalyst systems of CZA and CZZ in combination with HZSM-5 under similar reaction conditions: at 533 K, 50 bar and a syngas mixture CO_2_/H_2_/N_2_ of 3/9/1 (*cf.*Table 3), the reported DME productivities were approx. 250 g_DME_ (kg_cat_ h)^−1^ with CZA/HZSM-5 and 190 g_DME_ (kg_cat_ h)^−1^ with CZZ/HZSM-5. In our experiments, at 523 K, 50 bar and with a CO_2_/CO_*x*_ inlet-ratio of 1.00, the CZZ/FER system achieves a DME productivity of 421 g_DME_ (kg_cat_ h)^−1^ (MeOH catalyst specific), demonstrating the particular suitability of continuously co-precipitated CZZ in combination with FER.

### Influence of temperature and CO_2_/CO_*x*_ inlet-ratio on selectivity

**Fig. 4:**
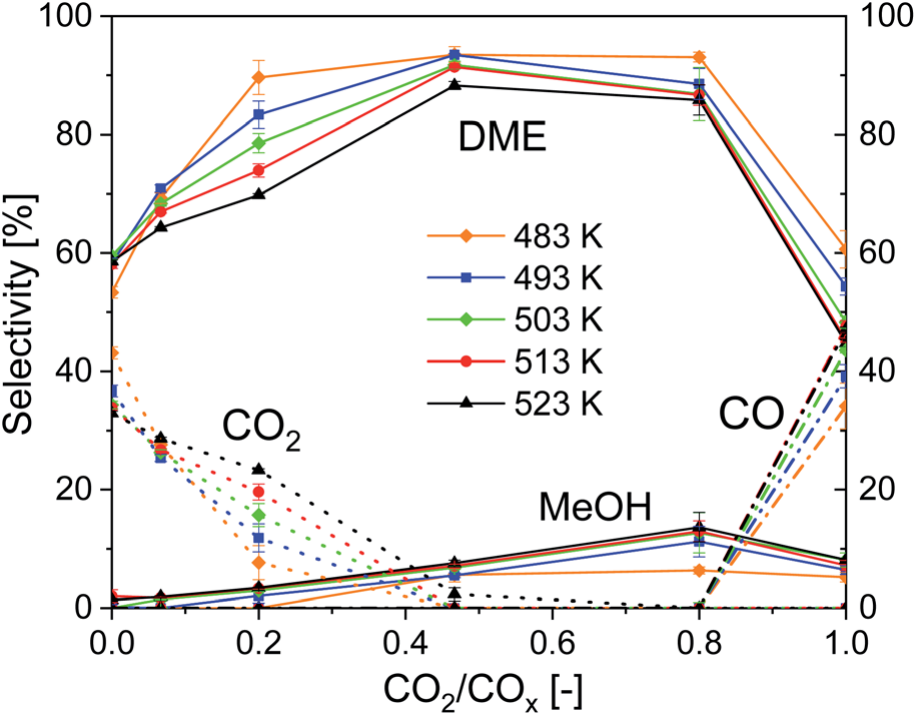
Influence of temperature and CO_2_/CO_*x*_ inlet-ratio on the selectivity, 18 000 ml_N_ (g h)^−1^, 50 bar, CZZ/FER 1 : 1 wt%.

Fig. 4 shows the influence of CO_2_/CO_*x*_ inlet-ratio and temperature on DME, MeOH, CO and CO_2_ selectivity using CZZ/FER. This diagram complements Fig. 3a, as it points out the influence of the feed composition on the selectivity of the four main carbon-containing species. At CO-rich feed compositions, CO_2_ is formed *via* the exothermic WGS (Case 2), resulting in a maximum CO_2_ selectivity of 43.1% (CO_2_/CO_*x*_ = 0.00) at the lowest measured temperature of 483 K. Increasing the amount of CO_2_ in the feed reduces the rate of WGS (R3), resulting in a decrease of CO_2_ selectivity, which in turn increases the selectivity to MeOH and DME. At a CO_2_/CO_*x*_ inlet-ratio of 0.47, both CO_2_ and CO are converted (Case 1), with increasing CO_2_ content the endothermic rWGS takes over and CO is formed (Case 3) with a maximum CO selectivity of 49.1% (CO_2_/CO_*x*_ = 1.00) at the highest measured temperature of 523 K. The impact of temperature on CO and CO_2_ selectivities, described before, leads to the respective differences in DME selectivity with changing temperature. The MeOH selectivity increases constantly from CO-rich feed compositions until a maximum at CO_2_/CO_*x*_ = 0.80 is reached. This can be attributed to the dehydration of MeOH to DME (R4), which can be negatively affected thermodynamically by higher water concentrations produced at higher CO_2_/CO_*x*_ inlet-ratios. Direct DME synthesis with feed gas compositions close to CO_2_/CO_*x*_ = 0.00 or 1.00 causes selectivity issues that might complicate an industrial process feasibility, as it would require an intensified CO/CO_2_ separation/recycling step.

Working with CO_2_ as sole carbon source lowers the thermodynamic equilibrium of CO_*x*_ conversion^41^ and reduces the efficiency of hydrogen use, since water is produced in a higher ratio compared to the valuable products (*i.e.* MeOH and DME).

Given the high DME selectivity, an average CO_2_/CO_*x*_ inlet ratio (*i.e.* approximately between 0.4 and 0.8) is not only a reasonable operating range within which both CO and CO_2_ are converted to DME, but it also offers the option of achieving a high DME productivity with a dynamic variation of the CO_2_/CO ratio.

### Catalyst stability

**Fig. 5:**
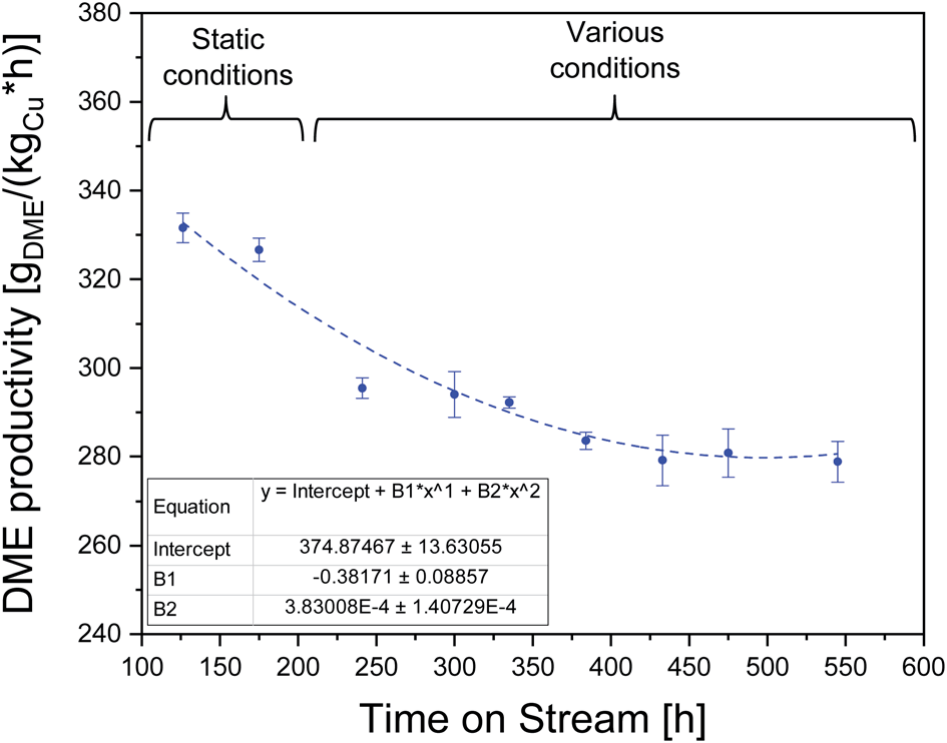
DME productivity between 126 and 545 h ToS at reference point: CO_2_/CO_*x*_ inlet-ratio = 0.8, 18 000 ml_N_ (g h)^−1^, 503 K, 50 bar, CZZ/FER 1 : 1 wt% with static reaction conditions in first 200 h ToS followed by various reaction conditions in temperature, CO_2_/CO_*x*_ inlet-ratio and GHSV. Dashed line fitted *via* OriginPro.

To assess the stability of the CZZ/FER catalyst, direct DME synthesis was operated over 550 h (Fig. 5). According to Fichtl *et al.*,^61^ the elevated water concentration formed in CO_2_-rich feed is the driving factor for irreversible deactivation effects. Therefore, and based on the above-mentioned arguments for a reasonable operating range, it seems appropriate to define a value of 0.80 for CO_2_/CO_*x*_ as reference point of the feed composition for this study.

For the period up to 200 h ToS, DME synthesis was performed under static reaction conditions, *i.e.* 503 K, 18 000 ml_N_ (g h)^−1^, CO_2_/CO_*x*_ = 0.80, 50 bar (reference point conditions). During this period, the activity of the catalyst in terms of DME productivity decreases to 326.6 g_DME_ (kg_Cu_ h)^−1^ (85.5%, 175 h) of the initial DME productivity (100%, 0–20 h ToS). Subsequently, the process was subjected to feed variation as described in Table 3 with 10 K temperature steps from 483 K to 523 K at five different GHSV between 18 000 and 42 000 ml_N_ (g h)^−1^ monitoring the recurring reference point after each variation cycle (Fig. S1†). DME productivity decreases to 283.6 g_DME_ (kg_Cu_ h)^−1^ (74.2%) up to a ToS of 384 h and remains almost unchanged at 278.9 g_DME_ (kg_Cu_ h)^−1^ (73.0%, 545 h) until the end of the observation period. DME selectivity was found to remain nearly constant, after a short run-in period of 5 h, with values in the range between 87.4 and 91.6%. This leads to the assumption that no relevant changes have taken place on the active sites of FER. Analogously, Frusteri *et al.*^12^ did not detect relevant coke formation working with CO_2_ as the sole carbon source. This can also be explained by the results of Sierra *et al.*^38^ who found that a slight increase in the water content in the gas phase reduces coke formation. Our results can confirm that relation: at CO-rich syngas concentrations and elevated temperatures, ethane was detected with CZZ/FER up to a maximum selectivity of 6.9% at 523 K, 50 bar, 36 000 ml_N_ (g h)^−1^ and a CO_2_/CO_*x*_ inlet-ratio of 0.00. Since a relatively low CO_*x*_ conversion range was achieved in the operating ranges considered, product concentrations were generally relatively low. Use of FER in a higher conversion range may result in increased formation of by-products such as methyl acetate, methane, ethane, and higher hydrocarbons. Hydrocarbon species were measured up to C_4_H_10_, concentrations below 0.01% by volume were not considered.

Our findings clearly demonstrate that the CZZ/FER catalyst is robust against fluctuations in the operating conditions after the initial operating phase and largely maintains its activity within the limits of the process parameter ranges investigated here.

## Conclusions

In this study, the admixed catalyst systems CZZ/FER, CZZ/γ-Al_2_O_3_ and CZA/FER were investigated in the direct DME synthesis from variable CO_2_/CO_*x*_ feeds. Our findings underline that a superior catalytic activity and a higher water resistance of a commercial FER-type zeolite clearly overtakes those of γ-Al_2_O_3_ leading to a consistent DME productivity applying different CO_2_/CO_*x*_ inlet-ratios. The effectiveness of FER occurs not only at high CO_2_/CO_*x*_ inlet-ratio but already at a slight increase of the CO_2_/CO_*x*_ ratio.

Combining a CZZ catalyst prepared by continuous precipitation method admixed with FER shows higher CO_*x*_ conversion and a significantly improved DME productivity for both, CO-rich feed (CO_2_/CO_*x*_ = 0.20, 1017 g_DME_ (kg_Cu_ h)^−1^) and CO_2_-rich feed conditions (CO_2_/CO_*x*_ = 1.00, 689 g_DME_ (kg_Cu_ h)^−1^) at 523 K, than the respective combination of a commercial CZA catalyst with FER. For CZZ/FER, we also found the option of adjusting DME productivity at 503 K largely independent of the CO_2_/CO_*x*_ ratio.

For CO_2_/CO_*x*_ inlet-ratios ranging between 0.47 and 0.80, temperatures between 483 K and 513 K and a GHSV of 18 000 ml_N_ (g h)^−1^, both CO_2_ and CO are converted – resulting in DME selectivities around 90%.

Detailed experiments with the CZZ/FER system performed under static and variable operating conditions showed that this catalytic system retains the major proportion of its initial DME productivity after 545 h time on stream. The over all deactivation in terms of DME productivity in the period from 0 to 545 h is 27.0%, and 14.6% during the period of variable feed conditions from 200 up to 545 h. The DME selectivity remains largely constant between 87.4% and 91.6% over the entire investigation duration. The extent to which aging phenomena due to sintering or coking play a role under process conditions is the subject of a planned investigation.

Our results prove the excellent suitability of CZZ/FER mixed catalyst systems for direct, flexible CO_*x*_ hydrogenation to DME under variable conditions. We believe that this type of catalyst system represents a promising option for use in sustainable power-to-fuel technologies that address both the use of hydrogen from renewable energy and the use of CO_2_ as a C_1_ raw material. For this reason, we are currently working intensively on modelling the process and optimising the composition of the catalyst bed and will report on this accordingly. Part of our work is furthermore to generate a sufficient data basis for a later planned kinetic modeling.

## Conflicts of interest

There are no conflicts to declare.

## Supplementary Material

RA-011-D0RA09754C-s001
