# Supplementary material for: Direct DME synthesis on CZZ/H-FER from variable CO2/CO syngas feeds
Source: RSC Adv. 2021 Jan 12;11(5):2556–64. doi: 10.1039/d0ra09754c (PMC8693869; doi:10.1039/d0ra09754c)
Supplement: RA-011-D0RA09754C-s001 [file RA-011-D0RA09754C-s001.pdf]

Electronic Supplementary Material (ESI) for RSC Advances.
This journal is © The Royal Society of Chemistry 2020

Supplementary Information

Stefan Wild, [a] Sabrina Polierer, [a] Thomas Zevaco, [a] David Guse, [b] Matthias Kind, [b] Jörg Sauer, [a] Stephan Pitter, [a] and Karla Herrera Delgado*[a]

[a.] *IKFT - Institute of Catalysis Research and Technology, Karlsruhe Institute of Technology, Hermann-von-Helmholtz-Platz 1, D-76344 Eggenstein-Leopoldshafen, Germany. E-mail: karla.herrera@kit.edu*

[b.] *TVT - Institute of Thermal Process Engineering, Karlsruhe Institute of Technology, Kaiserstraße 12, D-76131 Karlsruhe, Germany.*

The carbon balance was calculated according to equation S1†.

$$C - balance = \frac{\dot{n}_{CO,in} + \dot{n}_{CO_2,in} - (\dot{n}_{MeOH,out} + 2 * \dot{n}_{DME,out} + \dot{n}_{CO,out} + \dot{n}_{CO_2,out} + \nu_x \dot{n}_{C_xH_y})}{\dot{n}_{CO,in} + \dot{n}_{CO_2,in}} \quad \text{(S1)}$$

The measuring procedure with purging and reference point repetition is shown in Fig. S1†.

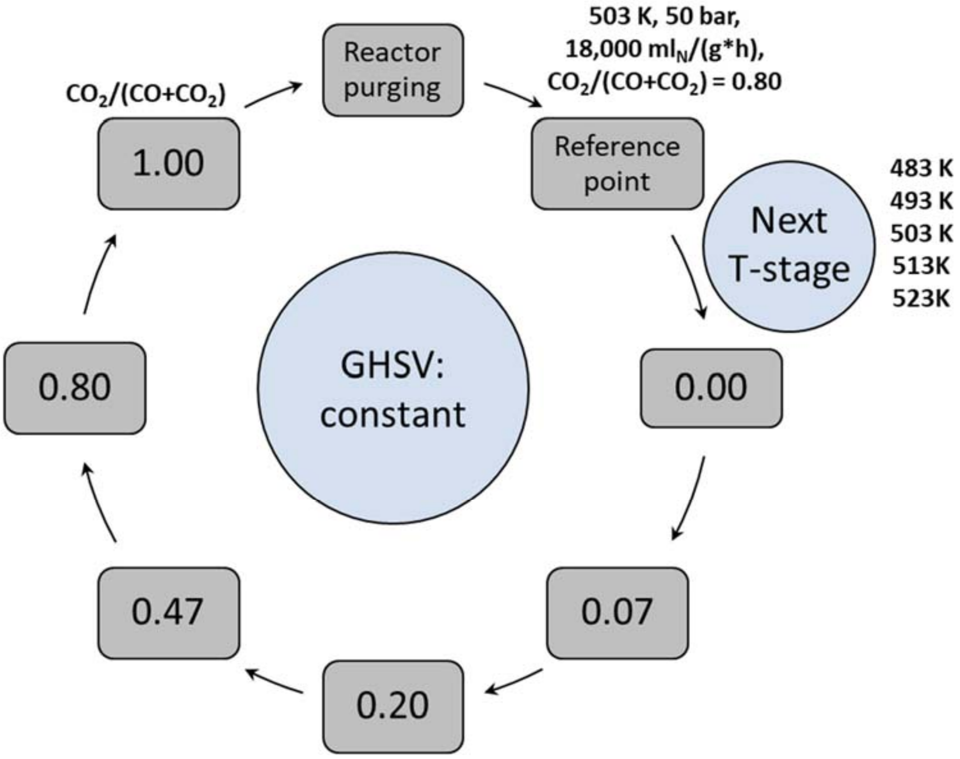

Fig. S1: Schematic illustration of the procedure of varying reaction conditions at a constant GHSV with recurring reference point at 50 bar.

All reaction conditions in this work were strongly, kinetically controlled due to the large distance to equilibrium (Fig S2†), exemplified by the CO_x conversion at the reaction conditions shown in main manuscript (Figure 2 a).

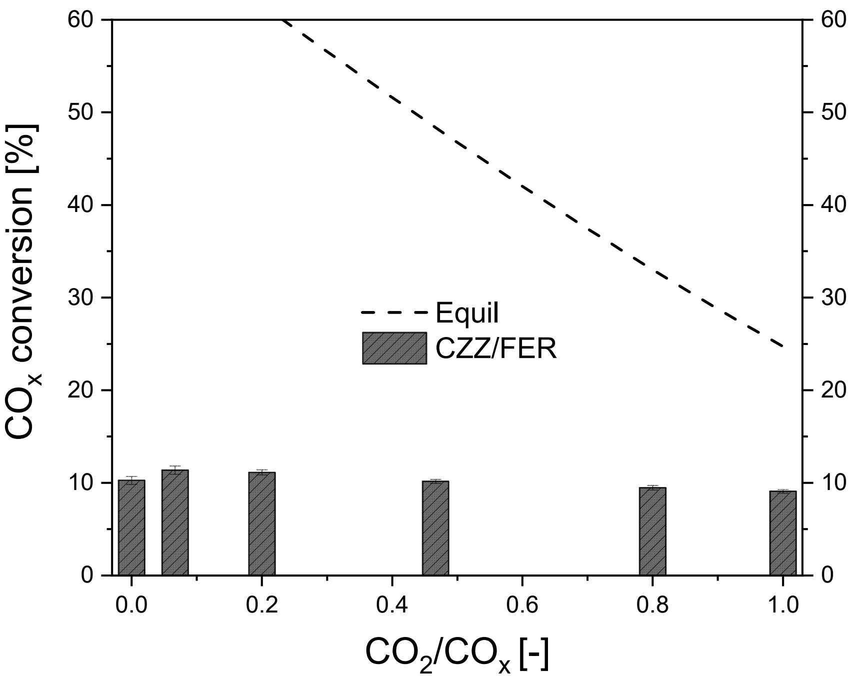

Fig. S2: CO_x conversion with CZZ/FER (1:1 wt%) (black bars) under variation of the CO_2/CO_x inlet ratio at 50 bar, 523 K and 36 000 mlN/(g·h).and the respective equilibrium curve calculated via ASPEN PLUS.

An overview of the influence of the CO_2/CO_x inlet-ratio on selectivity, CO/CO_2 conversion and MeOH/DME productivity can be found in Table S1†.

Table S1: Effect of CO_2/CO_x inlet-ratio with the use of CZZ/FER 1:1 wt% on the CO_2 and CO conversion, the DME, MeOH, CO_2 and CO selectivity, and the DME and MeOH productivity. Measurement conditions: GHSV: 18 000 $ml_N/(g*h)$, 50 bar and 523 K.

Inlet-ratio/ -	Conversion/ %		Selectivity/ %				Productivity/ $g_i/(kg_{Cu}*h)$	
CO_2/CO_x	CO_2	CO	DME	MeOH	CO_2	CO	DME	MeOH
0.00	<0	25.2	58.6[b]	1.4	32.9	<0	643.1	21.3
0.07	<0	26.9	64.3[b]	1.9	28.6	<0	684.1	28.1
0.20	<0	27.9	69.8[b]	3.4	23.3	<0	659.7	44.5
0.47	<0	30.0	88.3[b]	7.6	2.3	<0	586.2	69.8
0.80	13.2	15.4	85.8[a]	13.6	<0	<0	468.1	103.6
1.00	20.9	<0	42.3[c]	8.5	<0	49.1	364.0	101.3

a: Eq 5, b: Eq 6, c: Eq 7
